# Impact of Indoor Residual Spray with Synthetic Pyrethroid in Gandhinagar District, Gujarat

**DOI:** 10.4103/0970-0218.58384

**Published:** 2009-10

**Authors:** Mamta Dattani, PB Prajapati, Dinkar Raval

**Affiliations:** Malaria Branch, Health Department, Gandhinagar, India

**Keywords:** Annual parasitic incidence, indoor residual spray, malaria

## Abstract

**Background:**

Indoor residual spray (IRS), with appropriate insecticide, is an effective weapon for the control of malaria. Two rounds of indoor residual spray, with synthetic pyrethroid, are given in highly malaria endemic areas. It aims to prevent transmission of malaria by adult vector mosquitoes.

**Aims:**

To assess the impact of indoor residual spray in the highly malaria-endemic villages of Kalol taluka in Gandhinagar district.

**Design:**

High risk population for malaria, based on last three-year malaria situation.

**Setting:**

Malaria endemic rural areas in Gandhinagar district where indoor residual spray was undertaken with synthetic pyrethroid in 2006 and 2007.

**Study Variables:**

*Exploratory* - Rural areas; *Outcome* - coverage, acceptance.

**Analysis:**

Percentage and proportions.

**Results:**

Prior to the introduction of synthetic pyrethroid, in 2005, the annual parasitic incidence of the sprayed villages was 33.4. It came down to 8.8 in 2006. Continuation of this strategy in the same villages further brought down the annual parasitic incidence to 1.5 in 2007. A similar trend of steady decline was observed in actual numbers of cases and other malariometric indices as well.

**Conclusion:**

IRS, it still has a major role in the control of malaria if implemented with proper supervision, better coverage and community participation.

## Introduction

Gujarat is considered one of the malaria-endemic states in the country.(1) However, the state has been able to contain the problem of malaria quite successfully, since 2004, due to the sustained efforts and good monitoring mechanism put in place. Annual parasitic incidence here was 4.8 in 2004 and came down to 1.2 in 2007.(2)

Gandhinagar district has four talukas with a total population of 15.47 lakh. Of the four talukas, the problem of malaria was more in Kalol taluka, which lies towards the west, adjoining Mehsana and Ahmedabad districts. It had been a low endemic district, as far as malaria was concerned, till the year 2004. However, there was a focal outbreak of malaria in 22 villages of Kalol taluka during 2005. The annual parasitic incidence of the taluka has increased from 2.22 in 2004 to 8.41 in 2005. This taluka is largely plain. Augmentation of irrigation facilities has taken place due to the canal net work established under the Sardar Sarovar Project. This taluka receives moderate rain fall but during 2005 (1369mm), 2006(1060 mm) and 2007(1099 mm) heavy rain fall occurred. The major agricultural crops are wheat, paddy and seasonal vegetables. There are 70 villages in the taluka. The literacy rate ranges from 78 - 89%.

Under National Vector Borne Disease Control Programme (NVBDP) the accepted strategy is early diagnosis and complete treatment (EDCT) and integrated vector management. The emphasis here is to go for a mix of strategies to achieve optimum results. Integrated vector management comprises of several vector control options of which Indoor Residual Spray (IRS) with effective insecticide is the mainstay in the rural areas. Malathion 25% was used for undertaking IRS till 2006. The findings of entomological studies revealed the development of resistance among vector population to Malathion and therefore alternate and effective insecticide Synthetic Pyrethroid (Deltamethrine 2.5%) was chosen in subsequent years.(3) The villages, for undertaking IRS, were selected based on the level of malaria endemicity for the preceding three years as per the criteria developed by Government of India under malaria action program 1995.(4) A total of 23 villages were selected to be covered under regular rounds of spray during the peak of the transmission period.

This study was undertaken with an objective to assess the impact of IRS in highly malaria endemic villages of Kalol taluka of Gandhinagar district.

## Materials and Methods

The study area was Gandhinagar district located on the central part of Gujarat. The analysis of village wise malaria situation during 2003, 2004 and 2005 revealed that 23 villages located in Hajipur and Rancharda PHCs of the taluka contributed 62% of the total malaria incidence in the taluka. These villages were selected to be covered under IRS and in 2005, 2006 and 2007 this activity was implemented in the selected villages. During 2006 and 2007 the decision to change insecticide in the district was taken due to the development of resistance to Malathion (70% mean mortality rate as per WHO test paper conducted in 2005), poor acceptance to Malathion in the community, less epidemiological and entomological impact and accordingly Deltamathrine 2.5 W.P was chosen.(5) The required quantity of insecticide was provided by the state. The state follows a very stringent quality control mechanism for ensuring quality of the insecticide. The samples of the batches received at the district level are also drawn and sent for the laboratory analysis to confirm the quality. Spray strategy was finalized in the month of April in both years and spray programme was chalked out.

Intimation slips were distributed to each household covered under spray to inform them about actual date of spray with an intention to improve coverage. The required quantity of insecticide for each of the villages was made available at the respective village *panchayats*. Moreover, the insecticide was packed in sachets of 200 gms so that the required quantity (400 grams in 10 liters of water) is used for preparing suspension for spray. For concurrent supervision the staff working at peripheral viz. MPHW, MPHS, Laboratory Technician, FHWs, and FHS were trained and deployed at the respective villages and they were assigned with specific tasks related with supervision.

Village *panchayats* were informed from the district and taluka level about the actual date of spray. *Shibirs* were organized prior to spray in all the villages in coordination with NGOs. More over effort was made to reach different sections of the community by organizing *shibirs* in different *faliyas* of the village. The spray personnel were selected at the district level and they were provided with required spray equipments, protective garments and hands on training regarding use of spray equipments and monitoring of discharge rate of the stirrup pump being used on a daily basis.

To make supervision more effective the officers of the PHC, Block and District level were given checklist for the concurrent as well as consecutive supervision. And the discrepancies found during such visits were addressed immediately.

A map of Gandhinagar district and Kalol taluka is attached in image file as Map 1 and 2

The actual spray operation was undertaken in the selected villages as per the prescribed schedule as shown in the following Table 1 and PHC wise house coverage and room coverage achieved during spray in 2006 and 2007 is shown in Table 2.

**Table 1:** Summary of spray strategy finalized and implemented during 2006-2007

PHC	Year	Rounds	Villages covered	Population covered	Date of commencement and completion (First round)	Date of commencement and completion (Second round)
Rancharda	2006	2	16	34369	16/5/2006 - 3/6/2006	1/8/2006 -14/8/2006
Hajipur		2	6	19672	16/5/2006 - 3/6/2006	1/8/2006 -14/8/2006
Rancharda	2007	2	16	35230	1/5/2007 - 22/5/2007	16/7/2007 -27/7/2007
Hajipur		2	7	22455	13/5/2007 - 22/5/2007	28/7/2007 -3/8/2007

**Table 2:** Coverage achieved during spray in 2006 and 2007

Year	PHC	Round	Houses			Rooms		
				
			Targeted	Covered	Percentage	Targeted	Covered	Percentage
2006	Rancharda	1	6873	5994	87.2	18216	15884	87.2
	Hajipur	1	3934	3401	86.4	10426	9013	86.1
	Rancharda	2	6873	5858	85.2	18216	15534	85.2
	Hajipur	2	3934	3297	83.8	10426	8727	83.7
2007	Rancharda	1	7046	5993	85.1	18672	15881	85.0
	Hajipur	1	4491	3846	85.6	11901	10192	85.6
	Rancharda	2	7046	6139	87.1	18672	16079	86.1
	Hajipur	2	4491	3847	85.7	11901	9844	82.7

During 2006,2007 spray was undertaken as per schedule in all the villages as can be seen from Table 1. Room coverage achieved was above the desired 80%. During 2006 the total quantity of insecticide consumed was 2232 kg which gives a per capita consumption of 0.021 kg while in 2007 the insecticide consumed was 2380 kg insecticide and per capita usage was 0.023 kg.

For effective supervision, supervisory staff, from various levels were deployed as shown in Table 3

**Table 3:** Supervisory staff deployed during 2006 and 2007 for IRS

Year	MPHW	FHW	MPHS	MS	ADMO	Other staff	Total
2006	7	12	2	1	1	10	33
2007	12	11	2	1	1	13	50

In addition to the above, a team from the district level visited the villages covered under spray on a daily basis for supervision as well as to take corrective steps as and when required. Checklists were filled up by MOPHCs, BHOs, DMO, EMO, DPC, IECO, CDHO and RDD. During 2006, 22 duly filled checklists were received while in 2007 about 37 checklists were received. No major discrepancies were observed as per the checklist. In addition to IRS routine control and preventive measures like case detection and treatment through surveillance agencies, anti larval measures, biological control, source reduction, treatment with insecticide of community owned bed nets and awareness campaign were also undertaken in the high risk villages. *Shibirs* were organized in coordination with NGO called *Udgam* in each of the *faliyas*. Synergy with other programmes was also ensured for promoting community awareness. In each village messages for community awareness were transmitted during the course of Krushimahotsav and also through loud speakers.

One of the novel concepts put in to practice in the high risk villages was the adoption of these villages by the Medical Officers in the district. The Medical Officers along with their team visited the high risk villages at periodic intervals to implement the control and preventive activities and also to monitor and supervising activities like IRS. This focused approach with added emphasis on monitoring has also resulted in the visible impact on morbidity due to malaria in the high risk villages.

The objective of IRS is to curtail the transmission of malaria and therefore it is very essential to assess the impact this activity on the disease burden in the community. A well established surveillance mechanism is in place in all the villages covered under spray. The ABER of the PHCs covered under spray during 2006 and 2007 is more than 10% which indicates that the surveillance has been quite satisfactory.

**Figure 1:**
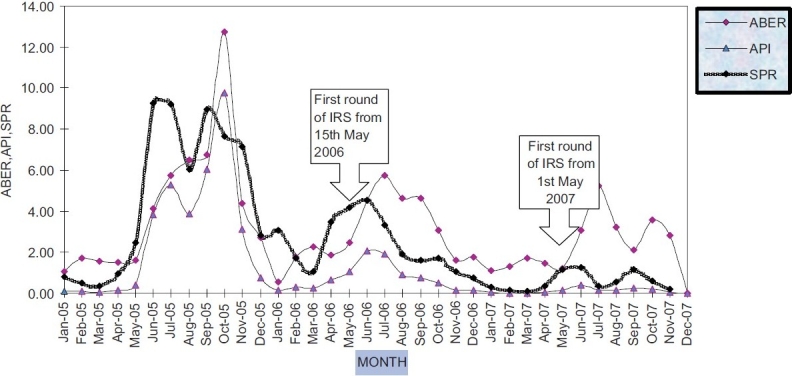
Monthwise malaria situation in insecticide sprayed villages from 2005-2007

Both the PHCs where the spray was undertaken had trained laboratory technician to examine the blood smear collected through the surveillance agencies. The malaria cases detected was monitored on a regular basis and the trend analysis of total malaria incidence over a period of three years in the sprayed villages shows a very good impact as regards reduction in number of cases. This is very well elaborated in the Figure 1.(6)

The Annual Parasitic Incidence in the 23 high risk villages was 33.4 in 2005 prior to the introduction of Synthetic Pyrethroid, which declined to a significantly low level to 8.8 in 2006 and 0.5 in 2007. This clearly shows the impact of Synthetic Pyrethoid spray.

Comparison of the impact of IRS in near by PHCs of the adjoining district, Mehsana has revealed a similar pattern of overall decline in the incidence of malaria during 2006 and 2007 (years sprayed with Synthetic Pyrethroid) as compared to 2005 (year sprayed with Malathion 25%).(7) But a closer analysis clearly depicts that the decline is not as significant as observed in Gandhinagar district as per the details given in the following Table 4.

**Table 4:** Comparison of the impact of IRS on malaria situation in Gandhinagar and Mehsana district

Year	Gandhinagar				Mehsana			
		
	API	SPR	SFR	% decline in comparison to previous year (API)	API	SPR	SFR	% decline in comparison to previous year (API)
2005	33.5	6.6	1.01	-	17.2	2.28	0.13	-
2006	8.8	2.5	0.16	73.73	8.25	1.46	0.02	52.03
2007	1.5	0.5	0.05	82.95	2.72	0.76	0.03	67.03

The percentage reduction in malaria incidence in the years when sprayed with Synthetic Pyrethroid was much more significant in Gandhinagar district as compared to Mehsana district which can be attributed to better supervision and monitoring of the spray activities on a day to day basis, in the villages covered. But in both the areas use of Syntetic pyrethroids has been effective in containing the transmission.

Periodic entomological studies undertaken in the sprayed areas during 2006 revealed Mean Mortality Rate (MMR) of vector mosquitoes on cement, wooden and mud surfaces around 85 to 90% indicating the biological efficacy of the insecticide.(8)

The age and sex wise classification of malaria cases detected in the high risk villages covered under spray during, 2006 and 2007 as compared to 2005 has revealed that that the proportion of malaria cases in children (0-15 years) has declined from 34% in 2005 to 19% in 2007 which is really a positive outcome. Similarly the proportion of malaria cases in females which was 42% in 2005 declined to 33% in 2007. This is mainly because women and children normally sleep indoors and due to the impact of spray malaria incidence in this vulnerable group declined significantly while the male population who sleep outdoor was exposed to mosquito bites.

Expenditure incurred for undertaking Indoor Residual spray during 2006 and 2007 is shown in the following Table 5.

**Table 5:** Expenditure incurred for IRS (Rs.)

Year	2006	2007
Population covered	108082	115370
Expenditure incurred (in Rs)	609072	760674
Expenditure per capita (In Rs)	5.6	6.6

The expenditure for undertaking spray in these high risk villages was 5.6 per capita in 2006 and 6.6 per capita in 2007, i.e. the expenditure to spray one house was only 30 to 40 rupees in a year which seems to be cost effective and is comparable with other methods also.

## Conclusions

The IRS undertaken in the 23 high risk villages of Kalol taluka in Gandhinagar district with Synthetic Pyrethroid during 2006 and 2007 as intervention measure for control of malaria has given significant impact on total malaria incidence and API. The API which was 33.43 in 2005 declined to 8.80 in 2006 (71 % reduction of %). The parasite incidence was 1.5 in 2007 which indicates the significant impact of IRS on malaria transmission. This impact could be achieved with the minimum cost of 5 to 6 rupees per capita. This study has clearly proved that, in spite of the constraints associated with IRS, it still has a major role in the control of malaria if implemented with proper supervision, better coverage and community participation. Though the epidemiological impact was quite evident, the entomological impact could not be ascertained due to insufficient data pertaining to the sprayed villages and therefore studies should be undertaken to monitor the density of vector species also to see the impact of vector control tools directly on vectors as well in such situations.(8) The NVBDCP in Gandhinagar district ensured that IRS is implemented in an effective manner and therefore the impact and results are quite evident.

**Figure 2:**
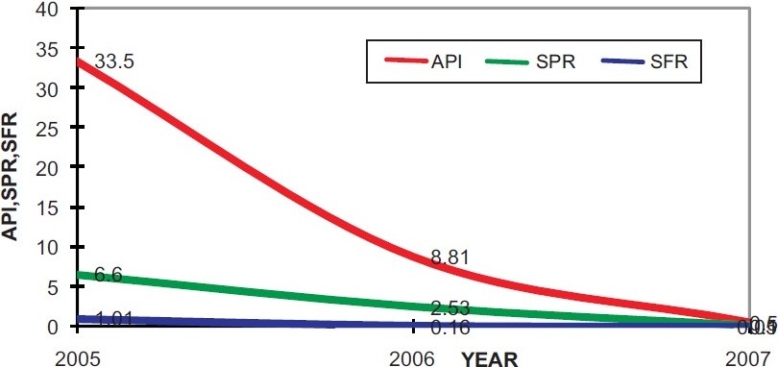
API, SPR and SFR of the problematic villages 2005 - 2007
